# Different dosing regimens for chronic knee osteoarthritis (KOA) pain management: A pooled analysis on celecoxib

**DOI:** 10.1007/s40520-025-03302-2

**Published:** 2026-01-23

**Authors:** Ernest Choy, Nicholas Fuggle, Egbert Biesheuvel, Srinivasan Venugopal, Sagar Suresh Kumbhar, Raffaella Maria Rita Chiaese, Chris Walker, Jean-Yves Reginster

**Affiliations:** 1 Cardiff Regional Experimental Arthritis Treatment and Evaluation (CREATE) Centre, Section of Rheumatology, Division of Infection and Immunity, School of Medicine, Cardiff University, Wales, UK; 2 MRC Lifecourse Epidemiology Centre, University of Southampton, Southampton, UK; 3 Biometrics, Viatris Inc, Amstelveen, Netherlands; 4 Biometrics, Viatris, Hyderabad India; 5 Biometrics, Viatris Inc, Bangalore, India; 6 Global Medical Affairs, Viatris, Milan, Italy; 7 Global Medical Affairs, Viatris, UK; 8 Protein Research Chair, Biochemistry Dept, College of Science, King Saud University, Riyadh, Kingdom of Saudi Arabia

**Keywords:** Celecoxib, Osteoarthritis (OA), Pain reduction, Moderate pain, Severe pain, Randomized controlled trials, Dosage regimen

## Abstract

**Background:**

Celecoxib is widely used for the management of different chronic musculoskeletal conditions including osteoarthritis (OA), but the comparative effectiveness of 200 mg once daily (OD) versus 100 mg twice daily (BID) in patients with varying baseline pain severity is not fully established.

**Aims:**

To compare the efficacy of celecoxib 200 mg OD and 100 mg BID in reducing pain among OA patients with moderate or severe baseline pain, using pooled post hoc analyses of two similar randomized controlled trials.

**Materials and methods:**

Data from two 6-week, double-blind, placebo-controlled trials in knee OA (*n* = 1,360) were pooled. Patients were stratified into moderate (VAS 40–69 mm, *n* = 675) or severe (VAS ≥ 70 mm, *n* = 685) pain subgroups. Interventions included celecoxib 100 mg BID, celecoxib 200 mg OD, or placebo. Primary endpoint was change from baseline in VAS pain at weeks 2 and 6, analyzed via mixed-effects model for repeated measures (MMRM) and ANCOVA with last observation carried forward. WOMAC pain score was a secondary endpoint.

**Results:**

Both celecoxib regimens significantly reduced VAS pain scores versus placebo at weeks 2 and 6 in the overall and moderate pain groups (*p* < 0.05). In severe pain patients, both regimens were superior to placebo at week 2; however, at week 6, only the 200 mg OD regimen retained statistical significance (LS mean difference vs. placebo − 7.45, *p* = 0.0135), while 100 mg BID did not. WOMAC pain score results mirrored VAS findings, with 200 mg OD showing the greatest improvement in severe baseline pain.

**Conclusion:**

Celecoxib 100 mg BID and 200 mg OD are both effective for OA pain relief, in moderate and severe pain. Findings suggest 200 mg OD may confer an advantage in patients with severe baseline pain in the long-term treatment (week 6).

**Supplementary Information:**

The online version contains supplementary material available at 10.1007/s40520-025-03302-2.

## Introduction

 Osteoarthritis (OA) is the most common form of arthritis worldwide, primarily impacting individuals over the age of 55 [1], and most frequently affecting the knees, hips, and hands [2]. Pain is usually the symptom with which patients initially present to their physician [3, 4].

The disease has no cure, and therefore symptom management is important as chronic pain (pain experienced for > 3 months) can ultimately lead to significant reductions in quality of life and disability in the long term [5, 6]. Chronic pain is now independently classified as a disease in its own right [7] and chronic pain has been shown to be associated with an increased risk of cardiovascular outcomes emphasising the importance of chronic pain’s effective management regardless of the underlying aetiology [8].

Guidelines for osteoarthritis management consistently recognize the importance of non-pharmacological interventions including patient education, weight management (where needed), and physical activity. As far as pharmacotherapy is concerned, whilst paracetamol was traditionally seen as an initial pharmacological intervention it’s efficacy in reducing pain is increasingly questioned in guidelines, particularly in the chronic phase of symptomatic disease [9, 10]. Oral nonsteroidal anti-inflammatory drugs (NSAIDs), including celecoxib are consistently recognized as providing an important role in the pharmacological management of OA, however their broad use is challenged by consideration of gastrointestinal and renal toxicities and cardiovascular risks presented by the patient’s history. The European Society for Clinical and Economic Aspects of Osteoporosis, Osteoarthritis and Musculoskeletal Diseases (ESCEO) recommends using NSAIDs judiciously at the lowest dose for the shortest duration necessary with celecoxib (200 mg/day) being preferred for its good short-term efficacy in OA at approved doses and its lower risk of toxicity, particularly at the GI level [10].

Whilst literature reporting on clinical trials for celecoxib supports the equivalence of OD and BID dosing (total daily dose of 200 mg/day), reinforcing the adaptability of the medicine as a therapy, based on patient preference and convenience [11–13]. The question of celecoxib’s efficacy in reducing pain in patients with different baseline pain severity has not been studied. This post hoc analysis of two 6-week osteoarthritis trials of very similar design using the same treatment interventions aims to assess whether the two different dosing regimens of 200 mg OD and 100 mg BID are equally effective in treating baseline OA pain self-reported as either moderate or severe [11, 12].

## Materials and methods

### Data set

This pooled analysis is based on two very similar randomised, double-blind, placebo-controlled company-sponsored studies (NH49-96-02-060 and NH49-98-02-087) conducted in 50 sites in the United States of America [11, 12]. The only difference between trials was that Study NH49-96-02-060 provided supplementary pharmacokinetics analysis on mean plasma concentrations for a small subset of patients.

Both studies evaluated the efficacy and safety of 100 mg BID celecoxib and 200 mg OD celecoxib in patients with knee OA diagnosed, according to American College of Rheumatology [14] criteria (Table 1). Treatment duration was six weeks, with the endpoint considered at weeks 2 and 6 for analysis, based on Visual Analogue Scale (VAS) pain score and at week 6 for Western Ontario MacMaster University (WOMAC) index [15, 16]. Analysis included subjects stratified into two pain severity subgroups (moderate and severe) at baseline, according to a 0–100 mm point VAS pain scale. Patients with mild pain (*n* = 44), characterized by baseline VAS < 40 mm, were excluded to be consistent with the original exclusion criteria of the individual trials. Only efficacy data from moderate (VAS = 40–69 mm) and severe (VAS ≥ 70 mm) subgroups were analyzed.

**Table 1:** Celecoxib clinical studies considered for the pooled analysis. VAS: visual analogue Scale; WOMAC: Western Ontario Mac master university index

Trial	Treatment arms	Participants	Completed	Duration	Efficacy parameter	Study Design
(study number)		(n)	(n)	(weeks)		
Williams GW, et al. J Clin Rheumatol. 2000;6(2):65–74 (**N49-96-02-060**)	100 mg celecoxib BID	231	194	6	VAS	Double-blind, placebo- controlled, parallel-group, multicenter study
	200 mg celecoxib OD	223	182		WOMAC	
	Placebo	232	146			
Williams GW, et al. Clin Ther. 2001;23(2):213 − 27 (**N49-98-02-087**)	100 mg celecoxib BID	243	194	6	VAS	Double-blind, placebo- controlled, parallel-group, multicenter study
	200 mg celecoxib OD	231	191		WOMAC	
	Placebo	244	164			

## Patients

Male and female subjects (≥ 18 years) meeting inclusion criteria, with Functional Capacity Classification between I and III [16] and in OA flare state, were included (Table 2). The populations studied in these two trials was a diverse representation of patients with OA and ~ 45% of the patients were over 65 years old at baseline (data not shown).

**Table 2:** Demographics characteristics FAS population according to baseline pain severity. Values are expressed as either numbers (%) of patients or mean ± Standard Deviation. VAS: visual analogue scale

	Moderate			Severe		
	Placebo	Celecoxib 100 mg BID	Celecoxib 200 mg OD	Placebo	Celecoxib 100 mg BID	Celecoxib 200 mg OD
	(*n* = 226)	(*n* = 232)	(*n* = 217)	(*n* = 238)	(*n* = 228)	(*n* = 219)
Sex						
Female	156 (69.03)	138 (59.48)	136 (62.67)	171 (71.85)	174 (76.32)	158 (72.15)
Male	70 (30.97)	94 (40.52)	81 (37.33)	67 (28.15)	54 (23.68)	61 (27.85)
Age (yr)	61.6 ± 12.0	62.0 ± 11.3	61.8 ± 12.3	62.4 ± 11.0	63.0 ± 11.1	62.1 ± 11.1
Height (cm)	167.5 ± 9.9	168.8 ± 9.9	168.2 ± 10.6	166.8 ± 9.9	166.1 ± 9.3	166.8 ± 10.1
Weight (Kg)	87.04 ± 17.7	88.53 ± 20.3	88.79 ± 18.6	91.86 ± 21.5	91.82 ± 23.1	94.46 ± 23.7
Ethnicity						
Asian	1 (0.44)	0	1 (0.46)	0	0	0
Black	16 (7.08)	19 (8.19)	14(6.45)	25 (10.5)	31 (13.6)	27(12.33)
Caucasian	204 (90.27)	206 (88.79)	193 (88.94)	204 (85.71)	188 (82.46)	181 (82.65)
Hispanic	5 (2.21)	6 (2.59)	6 (2.76)	8 (3.36)	8 (3.51)	11 (5.02)
Other	0	1 (0.43)	3 (1.38)	1 (0.42)	1 (0.44)	0

Patients were considered in an OA flare state if VAS ≥ 40 mm and Patient’s and Physician’s Global Assessment [17] scores (3 = fair; 4 = poor; 5 = very poor) were increased for one or more points from baseline visit. Subjects were randomised to receive either placebo, or celecoxib at the same dosage but different regimen (celecoxib 100 mg twice daily, or celecoxib 200 mg once daily) for 6 weeks. Subjects were instructed to take one capsule from Bottle A with breakfast and one capsule from Bottle B with the evening meal. Investigation drug supply scheme is provided in Supplementary Table 1.

## Outcome measures

The primary efficacy endpoint was the change from baseline in VAS pain score at week 6. Pain was assessed at baseline, week 2, and week 6. Secondary endpoints included changes in the Western Ontario and McMaster Universities Osteoarthritis Index (WOMAC) Pain score, assessed at baseline and week 6.

### Statistical analysis

All efficacy analyses were performed using the Full Analysis Set (FAS), consisting of all randomised patients who received at least one dose of study drug and had at least one post-baseline efficacy assessment. The primary endpoint was analysed using a mixed-effects model for repeated measures (MMRM) with study, treatment, visit, treatment-by-visit interaction, and baseline score as fixed effects. Additionally, an analysis of covariance (ANCOVA) was conducted using the Last Observation Carried Forward (LOCF) method for sensitivity analysis. Patients with missing data were accounted for using LOCF in the ANCOVA model. Subgroup analyses were conducted to compare the effects of celecoxib 100 mg BID and 200 mg OD within the moderate and severe pain groups. Least-Squares (LS) mean changes from baseline were calculated, and treatment differences were reported with 95% confidence intervals (CIs) and unadjusted p-values. Outputs were reviewed to validate model assumptions. Forest plots were generated to illustrate treatment effects at week 6 for the overall population and subgroups. All statistical analyses were performed using SAS version 9.4 (SAS Institute, Cary, NC, USA).

## Results

### Patients

The FAS had 1360 participants with 675 subjects in moderate pain group and 685 subjects in severe pain group. In the moderate pain group (VAS = 40–69), 226 received placebo, 232 received 100 mg celecoxib BID, and 217 received 200 mg celecoxib OD. In severe (VAS ≥ 70) pain group, 238 received placebo, 228 received 100 mg celecoxib BID and 219 received 200 mg celecoxib OD. Therefore, both subgroups are similar in sample size. Females were a majority in all groups with percentage ranging from 59.48% (moderate 100 mg BID) to 76.32% (severe 100 mg BID) of participants, while males ranged from 23.68% (severe 100 mg BID) to 40.52% (moderate 100 mg BID) of participants. Caucasians were the majority ethnic group, comprising 82.46% (severe 100 mg BID) to 90.27% (moderate Placebo). Mean age spanned 61.6 to 63.0 years, height 166.1 to 168.8 cm, and weight 87.04 to 94.46 kg, with slight variations. Other ethnic groups (Asian, Black, Hispanic, Other) had low representation. The demographic details are shown in Table 2.

## Efficacy

### Primary efficacy endpoint –VAS pain score

**Fig. 1:**
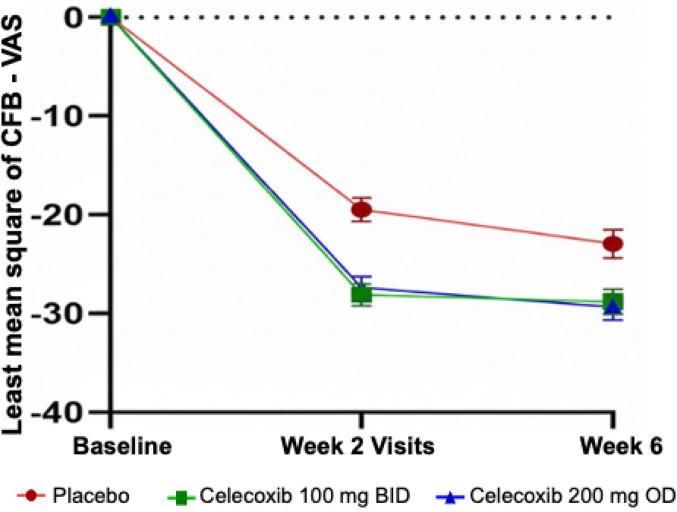
LSMeans (SE) Change from Baseline in VAS pain at week 2 and 6 in the FAS population; VAS = Visual Analog Scale; SE = Standard Error; CFB = Change From Baseline

The change from baseline in VAS pain scores was assessed at week 2 and week 6. All treatment groups exhibited a reduction in mean VAS pain scores from baseline. At week 2 and week 6, the reduction in VAS scores was significantly greater in both celecoxib treatment groups compared to placebo indicating improvement in OA pain symptoms as illustrated by the LSMeans (SE) in Fig. 1.

MMRM analysis of the least squares mean (SE) change from baseline in the VAS pain scores demonstrated a significantly greater reduction in patients receiving both 100 mg BID and 200 mg OD of celecoxib compared to placebo. At week 2, LS mean change from baseline difference (95% CI) was − 8.66 (–11.91, − 5.41) for the 100 mg BID group (*p* < 0.0001) and − 7.93 (–11.20, − 4.66) for the 200 mg OD group (*p* < 0.0001) compared to placebo. At week 6, this trend was sustained, with a LS mean change difference (95% CI) of − 5.88 (–9.66, − 2.10) for the 100 mg BID group (*p* = 0.0023) and − 6.48 (–10.29, − 2.66) for the 200 mg OD group (*p* = 0.0009) compared to placebo. Across the 6-week treatment period, ANCOVA analysis confirmed a significantly greater overall LS mean change from baseline in the active treatment groups compared to placebo. The LS mean change difference (95% CI) was − 6.43 (–10.06, − 2.81) for 100 mg BID (*p* = 0.0005) and − 6.90 (–10.56, − 3.25) for 200 mg OD (*p* = 0.0002), indicating consistent efficacy of both dosing regimens in reducing OA pain severity relative to placebo in patients with moderate and severe pain (Supplementary Table 2).

### Subgroup analysis by pain severity at baseline

**Fig. 2:**
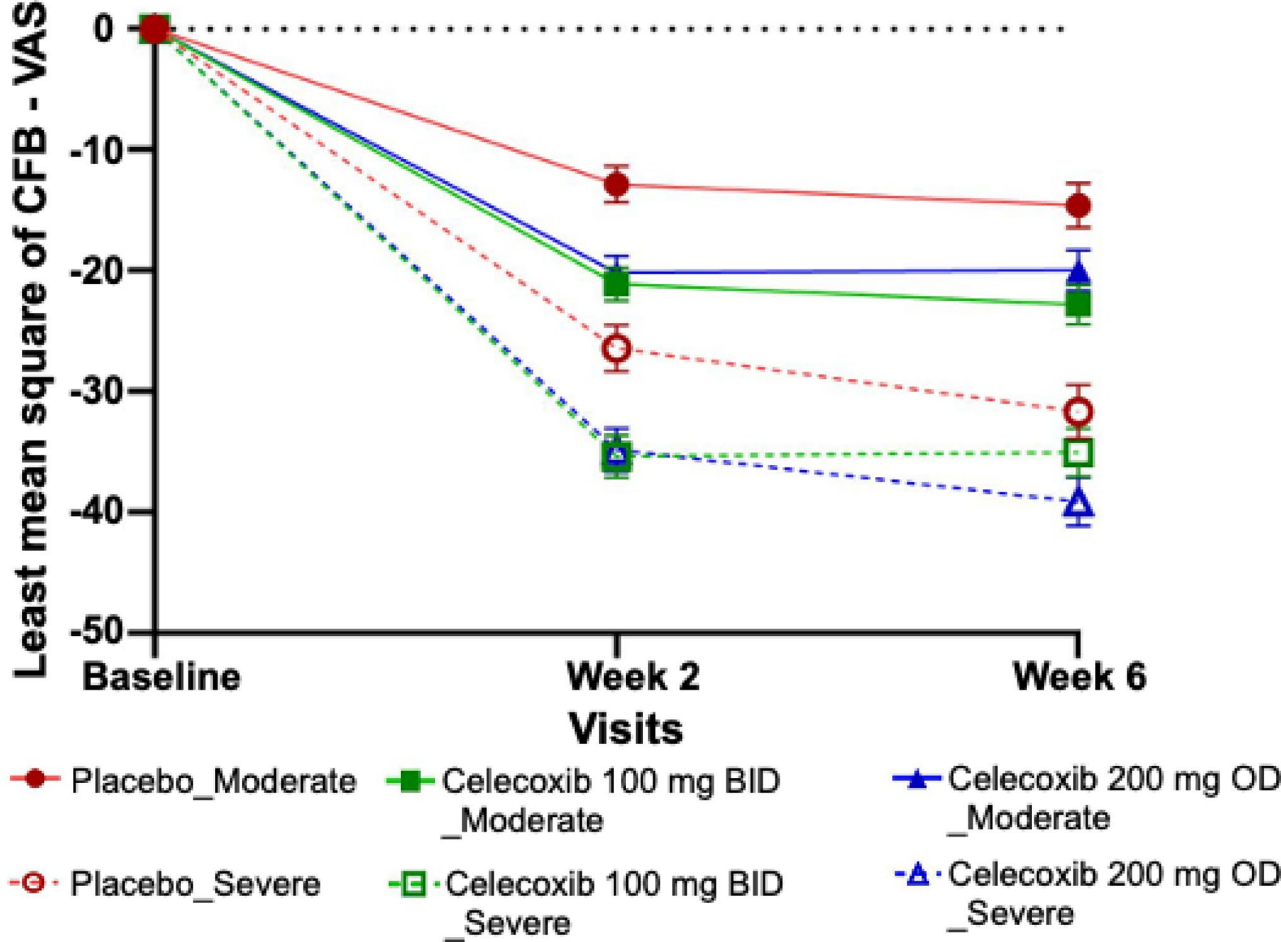
LSMeans (SE) VAS pain Change from baseline in VAS pain at week 2 and 6 in the FAS population, stratified by severity at week 2 and 6 in the FAS population. VAS = Visual Analog Scale; SE = Standard Error; CFB = Change From Baseline

Patients with either moderate or severe pain were independently analyzed as subgroups to compare the efficacy of both celecoxib treatment groups; at 2 weeks, both dosage regimens (100 mg BID and 200 mg OD) effectively reduced pain in both treatment groups when compared to placebo. At week 6, 100 mg BID and 200 mg OD had similar efficacy in moderate pain group, while 100 mg BID was less effective than 200 mg OD in severe pain group (Fig. 2).

Among patients with moderate pain, the MMRM analysis of the least squares mean (SE) change from baseline in VAS pain scores demonstrated a significantly greater reduction in patients treated with either 100 mg BID or 200 mg OD of celecoxib compared to those receiving placebo from week 2 onwards (Fig. 2).

At week 2, the LS mean change from baseline difference (95% CI) compared to placebo was − 8.25 (–12.27, − 4.23) for the 100 mg BID group (*p* < 0.0001) and − 7.35 (–11.42, − 3.27) for the 200 mg OD group (*p* = 0.0004). At week 6, similar results were seen, with a LS mean change difference (95% CI) of − 8.18 (–13.01, − 3.35) for the 100 mg BID group (*p* = 0.0009) and − 5.37 (–10.27, − 0.47) for the 200 mg OD group (*p* = 0.0318) compared to placebo. Over the 6-week treatment period, ANCOVA analysis demonstrated statistically significant greater change from baseline in both active treatment groups compared to placebo indicating consistent efficacy of both dosing regimens in reducing osteoarthritis pain severity in patients with moderate pain (Supplementary Table 3).

The severe subgroup showed numerically greater changes from baseline in VAS pain score in all treatment arms compared to the moderate subgroup, which is not unexpected as they had higher baseline values on average. In patients with severe pain, MMRM analyses of the least squares mean (SE) change from baseline in VAS pain scores revealed a pattern similar to that of the moderate subgroup, with the exception of the 100 mg BID group at week 6. At week 2, the LSMeans (SE) change from baseline difference (95% CI) at week 2 was − 8.96 (–14.13, − 3.79; *p* = 0.0007) for the 100 mg BID group and − 8.43 (–13.62, − 3.24; *p* = 0.0015) for the 200 mg OD group. At week 6 the LSMeans was also statistically significant for 200 mg OD celecoxib at −7.45 (−13.35, −1.54) (*p* = 0.0135), however, for the 100 mg BID group, the reduction was not statistically significant compared with placebo at −3.37 (−9.23, 2.49) (*p* = 0.2592) (Supplementary Table 4). The ANCOVA analyses confirmed the MMRM results, with a statistically significant reduction in pain only for the 200 mg group with LS mean change difference (95% CI) compared to placebo of −8.11 (−13.73, −2.49) (*p* = 0.0047), and a not statistically significant difference for the 100 mg BID group of −4.14 (−9.74, 1.45) (*p* = 0.1465) (Supplementary Table 4).

**Fig. 3:**
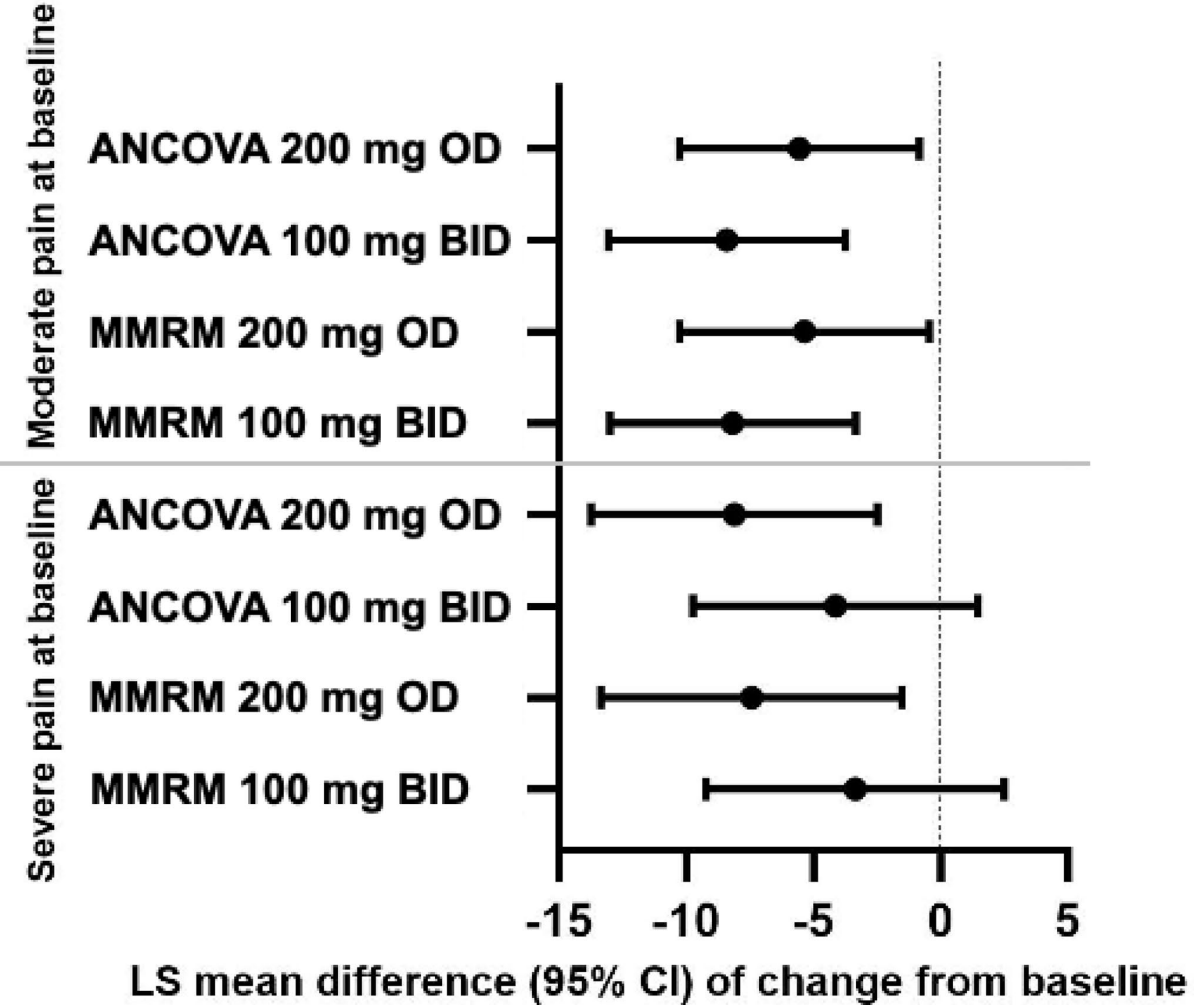
Forest Plot VAS Analysis at 6 weeks. LS mean difference and 95% CI are derived from MMRM (mixed-effects model for repeated measures) model including terms for study, treatment, visit, treatment-by-visit, and baseline score. An unstructured covariance matrix was used for the MMRM model. LS mean difference and 95% CI are derived from ANCOVA (analysis of covariance) model including terms for study, treatment, and baseline score, and using Last Observation Carried Forward (LOCF)

The forest plots of the VAS pain reductions versus placebo illustrate the consistency between the MMRM and ANCOVA analyses. These analyses demonstrated that both 100 mg BID and 200 mg OD doses of celecoxib yielded consistent and comparable results in the overall and moderate subgroup. In the severe subgroup, the 200 mg OD celecoxib demonstrated better efficacy than the 100 mg BID, as the 100 mg dose did not achieve statistical significance at 6 weeks of treatment (Fig. 3).

### Secondary efficacy endpoint – WOMAC pain score

Considering the WOMAC pain scale comparing baseline and week 6, all groups showed gradual improvement from baseline to week 6; however, the extent of improvement varied by treatment. The 200 mg OD celecoxib group demonstrated the largest mean change, with the most notable benefit observed in the severe subgroup, indicating superior efficacy in this population. The 100 mg BID celecoxib group demonstrated an intermediate level of improvement, providing benefit over placebo but less than the higher dose of 200 mg OD (Supplementary Tables 5,6,7; Supplementary Fig. 1). The results are similar on both VAS and WOMAC scales, suggesting 200 mg OD reduces pain most efficiently in patients with severe pain at baseline.

### Pharmacokinetics

Pharmacokinetic data were available only for study id N49-96-02-060. A 7 mL blood sample was taken - prior administration of the morning dose of study medication – from a small subset of patients (32 patients from the celecoxib 100 mg BID treatment group and 39 patients from the celecoxib 200 mg OD group), at week 2 and week 6 for determination of plasma concentrations of celecoxib. Plasma concentrations were higher in patients receiving celecoxib 200 mg OD. At week 2, the mean plasma concentrations of celecoxib were 178 ± 131 ng/mL for patients receiving 100 mg BID, and 284 ± 176 ng/mL for those receiving 200 mg OD. By week 6, these levels slightly decreased to 153 ± 85 ng/mL for the 100 mg BID group and 270 ± 187 ng/mL for the 200 mg OD group (Supplementary Table 8).

## Discussion

Celecoxib as a COX-2 selective NSAID, is used for the management of different chronic musculoskeletal conditions including osteoarthritis. The recommended dose for the treatment of OA is 100 mg BID or 200 mg OD [18]. In this post hoc pooled analysis of two very similar trials, we evaluated drug efficacy in reducing knee OA pain considering the two different dose regimens, in comparison with placebo.

When considering all patients pooled together across both studies, this analysis demonstrated that both celecoxib dosing regimens significantly reduced OA pain compared to placebo. Significant reduction in VAS pain from baseline was observed as early as week 2 and sustained through week 6. This is consistent with previous studies [19–21] highlighting that the majority of any treatment effect on VAS pain (also confirmed by WOMAC pain subscale) was evident within 2 weeks and the effect was sustained over the remainder of the study period.

Interestingly, the pooled analysis by severity, demonstrated a significant reduction in pain at week 6 for the once-a-day dosage, that was not observed with twice-a-day administration, in patients with severe baseline pain (VAS ≥ 70 mm). Notably this is inconsistent with the 2-week data by pain severity (both moderate and severe baseline pain) where both dosing regimens statistically separated from placebo and the results may therefore be down to the play of chance.

However, the additional pharmacokinetic data available evaluated from a small subset of patient’s clinical study (N49-96-02-060), consistently demonstrated the presence of a higher drug concentration with the 200 mg OD evening administration at both weeks 2 and 6, which could be a potential factor contributing to the results described.

A key limitation of this analysis is that the findings are drawn from a relatively small evidence base. In this analysis we relied on just two of a substantial number of OA clinical trials for celecoxib, which was justifiable given the near identical study design. Further prospective investigation of these dosing regimens is unlikely to occur, and most trials do not compare the two dosing regimens directly. At present only one other study [13] demonstrated that both dosages provide similar improvement according to WOMAC composite score and patient’s global assessment of OA although no analysis by baseline severity was conducted. Similarly, a network meta-analysis [22] indicated comparable efficacy with no statistically significant difference in pain control among the two dosage regimens [200 mg QD: SMD = − 0.38 (95% CI: −0.50 to − 0.27); 100 mg BID: SMD = − 0.42 (95% CI: −0.59 to − 0.24)].

In conclusion, the analysis presented here is largely consistent with the work of other authors [11–13] in suggesting that both 100 mg BID and 200 mg OD are effective strategies to treat osteoarthritis pain. The findings of this analysis confirm that celecoxib is an effective treatment for OA pain, with both dosing strategies offering significant benefit in pain reduction for the most part, with post hoc findings by baseline pain severity weakly inferring that the 200 mg OD dose may be a preferred treatment choice in patients with severe baseline pain.

### Limitations

This study is a post hoc pooled analysis of only two trials, which, although similar in design, limits the generalizability of findings. The pharmacokinetic data were derived from a small subset of participants and may not fully represent the broader study population. The study duration was limited to 6 weeks, so conclusions cannot be extended to long-term efficacy or safety.

## Supplementary Information

Below is the link to the electronic supplementary material.

Supplementary Material 1

## Data Availability

Data available from the corresponding author on reasonable request.

## Declarations

### Patient and public involvement

Patients or the public were not involved in the design, or conduct, or reporting, or dissemination plans of our research.
