# Supplementary material for: Different dosing regimens for chronic knee osteoarthritis (KOA) pain management: A pooled analysis on celecoxib
Source: Aging Clin Exp Res. 2026 Jan 23;38(1):55. doi: 10.1007/s40520-025-03302-2 (PMC12852159; doi:10.1007/s40520-025-03302-2)
Supplement: Supplementary file 1 — Supplementary Material 1 [file 40520_2025_3302_MOESM1_ESM.docx]

**SUPPLEMENTARY MATERIALS**

***Supplementary Tables***

**Supplementary Table 1** Investigational Group Supplies Scheme

|  | **Study Visit 1 (baseline)** | **Study Visit 2**  **(Week 2)** |
| --- | --- | --- |
| **Group 1 (100 mg celecoxib BID)** | | |
| *Bottle A*: one celecoxib 100 mg capsule AM | 17 capsules | 34 capsules |
| *Bottle B*: one celecoxib 100 mg capsule PM | 17 capsules | 34 capsules |
| **Group 2 (200 mg celecoxib OD)** | | |
| *Bottle A*: one placebo capsule AM | 17 capsules | 34 capsules |
| *Bottle B*: one celecoxib 200 mg capsule PM | 17 capsules | 34 capsules |
| **Group 3 (Placebo)** | | |
| *Bottle A*: one placebo capsule AM | 17 capsules | 34 capsules |
| *Bottle A*: one placebo capsule AM | 17 capsules | 34 capsules |

**Supplementary Table 2** Least-Squares mean (SE) change from baseline in VAS pain by Visit and Least-Squares mean difference at the end of 6 weeks for patients with moderate and severe pain at baseline

|  | | **Pooled Studies** | | | |
| --- | --- | --- | --- | --- | --- |
|  |  | **Placebo**  **(n=464)** | **Celecoxib 100mg BID**  **(n=460)** | **Celecoxib 200mg OD**  **(n=436)** | |
| Observed Mean (SD) | Baseline | 69.20 (14.65) | 68.77 (15.36) | 68.05 (15.18) | |
| LS Mean change from baseline (SE) by visit | Week 2 | -19.47 (1.22) | -28.13 (1.12) | -27.40 (1.14) | |
|  | LS mean difference (95% CI) |  | -8.66 (-11.91, -5.41) | -7.93 (-11.20, -4.66) | |
|  | p-value^a^ |  | <0.0001 | <0.0001 | |
|  | Week 6 | -22.95 (1.43) | -28.83 (1.29) | -29.43 (1.32) | |
|  | LS mean difference (95% CI) |  | -5.88 (-9.66, -2.10) | -6.48 (-10.29, -2.66) | |
|  | p-value^a^ |  | 0.0023 | 0.0009 | |
|  | | **Pooled Studies** | | | |
|  |  | **LS Means**  **(95% CI)** | **LS Means difference**  **(95% CI)** | | **p-value^b^** |
| Placebo (n=342) | | -22.32 (-24.99, -19.65) |  | |  |
| Celecoxib 100mg BID  (n=404) | | -28.75 (-31.21, -26.30) | -6.43 (-10.06, -2.81) | | 0.0005 |
| Celecoxib 200mg OD  (n=391) | | -29.22 (-31.72, -26.73) | -6.90 (-10.56, -3.25) | | 0.0002 |

LS = Least-Squares; SD = Standard Deviation; SE = Standard Error

^a^p-value derived from MMRM (mixed-effects model for repeated measures) model including terms for study, treatment, visit, treatment-by-visit, and baseline score. An unstructured covariance matrix was used for the MMRM model.

^b^p-value derived from ANCOVA (analysis of covariance) model including terms for study, treatment, and baseline score, and using Last Observation Carried Forward (LOCF).

**Supplementary Table 3** Least-Squares mean (SE) change from baseline in VAS pain by Visit and Least-Squares mean difference at the end of 6 weeks for patients with moderate pain at baseline

|  | | **Pooled Studies** | | | |
| --- | --- | --- | --- | --- | --- |
|  |  | **Placebo**  **(n=226)** | **Celecoxib 100mg**  **BID**  **(n=232)** | **Celecoxib 200mg**  **OD**  **(n=217)** | |
| Observed Mean (SD) | Baseline | 56.69 (8.52) | 56.20 (8.54) | 55.30 (8.22) | |
| LS Mean change from baseline (SE) by visit | Week 2 | -12.84 (1.51) | -21.09 (1.38) | -20.18 (1.42) | |
|  | LS mean difference (95% CI) |  | -8.25 (-12.27, -4.23) | -7.35 (-11.42, -3.27) | |
|  | p-value^a^ |  | <0.0001 | 0.0004 | |
|  | Week 6 | -14.62 (1.83) | -22.80 (1.64) | -19.99 (1.69) | |
|  | LS mean difference (95% CI) |  | -8.18 (-13.01, -3.35) | -5.37 (-10.27, -0.47) | |
|  | p-value^a^ |  | 0.0009 | 0.0318 | |
|  | | **Pooled Studies** | | | |
|  |  | **LS Means**  **(95% CI)** | **LS Means difference**  **(95% CI)** | | **p-value^b^** |
| Placebo (n=174) | | -14.32 (-17.75, -10.88) |  | |  |
| Celecoxib 100mg BID  (n=206) | | -22.73 (-25.88, -19.58) | -8.41 (-13.07, -3.75) | | 0.0004 |
| Celecoxib 200mg OD  (n=196) | | -19.87 (-23.10, -16.63) | -5.55 (-10.27, -0.83) | | 0.0213 |

LS = Least-Squares; SD = Standard Deviation; SE = Standard Error

^a^p-value derived from MMRM (mixed-effects model for repeated measures) model including terms for study, treatment, visit, treatment-by-visit, and baseline score. An unstructured covariance matrix was used for the MMRM model.

^b^p-value derived from ANCOVA (analysis of covariance) model including terms for study, treatment, and baseline score, and using Last Observation Carried Forward (LOCF).

**Supplementary Table 4** Least-Squares mean (SE) change from baseline in VAS pain by Visit and Least-Squares mean difference at the end of 6 weeks for patients with severe pain at baseline

|  | | **Pooled Studies** | | | |
| --- | --- | --- | --- | --- | --- |
|  |  | **Placebo**  **(n=238)** | **Celecoxib 100mg BID**  **(n=228)** | **Celecoxib 200mg OD**  **(n=219)** | |
| Observed Mean (SD) | Baseline | 81.08 (7.71) | 81.56 (8.79) | 80.68 (8.42) | |
| LS Mean change from baseline (SE) by visit | Week 2 | -26.42 (1.94) | -35.38 (1.78) | -34.85 (1.80) | |
|  | LS mean difference (95% CI) |  | -8.96 (-14.13, -3.79) | -8.43 (-13.62, -3.24) | |
|  | p-value^a^ |  | 0.0007 | 0.0015 | |
|  | Week 6 | -31.69 (2.22) | -35.06 (1.99) | -39.14 (2.02) | |
|  | LS mean difference (95% CI) |  | -3.37 (-9.23, 2.49) | -7.45 (-13.35, -1.54) | |
|  | p-value^a^ |  | 0.2592 | 0.0135 | |
|  | | **Pooled Studies** | | | |
|  |  | **LS Means**  **(95% CI)** | **LS Means difference**  **(95% CI)** | | **p-value^b^** |
| Placebo (n=168) | | -30.67 (-34.79, -26.56) |  | |  |
| Celecoxib 100mg BID  (n=198) | | -34.81 (-38.61, -31.02) | -4.14 (-9.74, 1.45) | | 0.1465 |
| Celecoxib 200mg OD  (n=195) | | -38.78 (-42.60, -34.96) | -8.11 (-13.73, -2.49) | | 0.0047 |

LS = Least-Squares; SD = Standard Deviation; SE = Standard Error

^a^p-value derived from MMRM (mixed-effects model for repeated measures) model including terms for study, treatment, visit, treatment-by-visit, and baseline score. An unstructured covariance matrix was used for the MMRM model.

^b^p-value derived from ANCOVA (analysis of covariance) model including terms for study, treatment, and baseline score, and using Last Observation Carried Forward (LOCF).

**Supplementary Table 5** Least-Squares mean (SE) change from baseline in WOMAC pain by Visit and Least-Squares mean difference at the end of 6 weeks for patients with moderate and severe pain at baseline

|  | **Pooled Studies** | | |
| --- | --- | --- | --- |
|  | **LS Means**  **(95% CI)** | **LS Means difference**  **(95% CI)** | **p-value^a^** |
| Placebo (n=298) | -2.57 (-2.97, -2.17) |  |  |
| Celecoxib 100mg BID  (n=376) | -3.46 (-3.81, -3.10) | -0.88 (-1.42, -0.35) | 0.0013 |
| Celecoxib 200mg OD  (n=355) | -3.55 (-3.91, -3.18) | -0.97 (-1.52, -0.43) | 0.0005 |

^a^p-value derived from ANCOVA (analysis of covariance) model including terms for study, treatment, and baseline score.

**Supplementary Table 6** Least-Squares mean (SE) change from baseline in WOMAC pain by Visit and Least-Squares mean difference at the end of 6 weeks for patients with moderate pain at baseline

|  | **Pooled Studies** | | |
| --- | --- | --- | --- |
|  | **LS Means**  **(95% CI)** | **LS Means difference**  **(95% CI)** | **p-value^a^** |
| Placebo (n=151) | -1.97 (-2.49, -1.44) |  |  |
| Celecoxib 100mg BID  (n=194) | -3.10 (-3.56, -2.63) | -1.13 (-1.83, -0.43) | 0.0016 |
| Celecoxib 200mg OD  (n=180) | -2.94 (-3.42, -2.46) | -0.97 (-1.69, -0.26) | 0.0076 |

^a^p-value derived from ANCOVA (analysis of covariance) model including terms for study, treatment, and baseline score.

**Supplementary Table 7** Least-Squares mean (SE) change from baseline in WOMAC pain by Visit and Least-Squares mean difference at the end of 6 weeks for patients with severe pain at baseline

|  | **Pooled Studies** | | |
| --- | --- | --- | --- |
|  | **LS Means**  **(95% CI)** | **LS Means difference**  **(95% CI)** | **p-value^a^** |
| Placebo (n=147) | -3.22 (-3.83, -2.62) |  |  |
| Celecoxib 100mg BID  (n=182) | -3.86 (-4.41, -3.31) | -0.64 (-1.45, 0.18) | 0.1267 |
| Celecoxib 200mg OD  (n=175) | -4.13 (-4.69, -3.58) | -0.91 (-1.74, -0.08) | 0.0308 |

^a^p-value derived from ANCOVA (analysis of covariance) model including terms for study, treatment, and baseline score.

**Supplementary Table 8** Summary of celecoxib mean plasma concentrations (ng/ml) at 2 and 6 weeks in a subset of patients with at least one dose of study (study N49-96-02-060) medication.

|  | **Celecoxib 100 mg BID (n=32)** | **Celecoxib 200 mg OD (n=39)** |
| --- | --- | --- |
| Week 2 |  |  |
| Mean (ng/ml) | 178.41 | 283.91 |
| SD (ng/ml) | 130.538 | 175.539 |
|  |  |  |
| Week 6 |  |  |
| Mean (ng/ml) | 152.88 | 270.37 |
| SD (ng/ml) | 84.815 | 187.407 |

SD = Standard Deviation

***Supplementary Figures***

**Supplementary Fig. 1** Change from Baseline (± SE) WOMAC pain score, stratified by Severity and Visit, for patients with moderate and severe pain at baseline

**
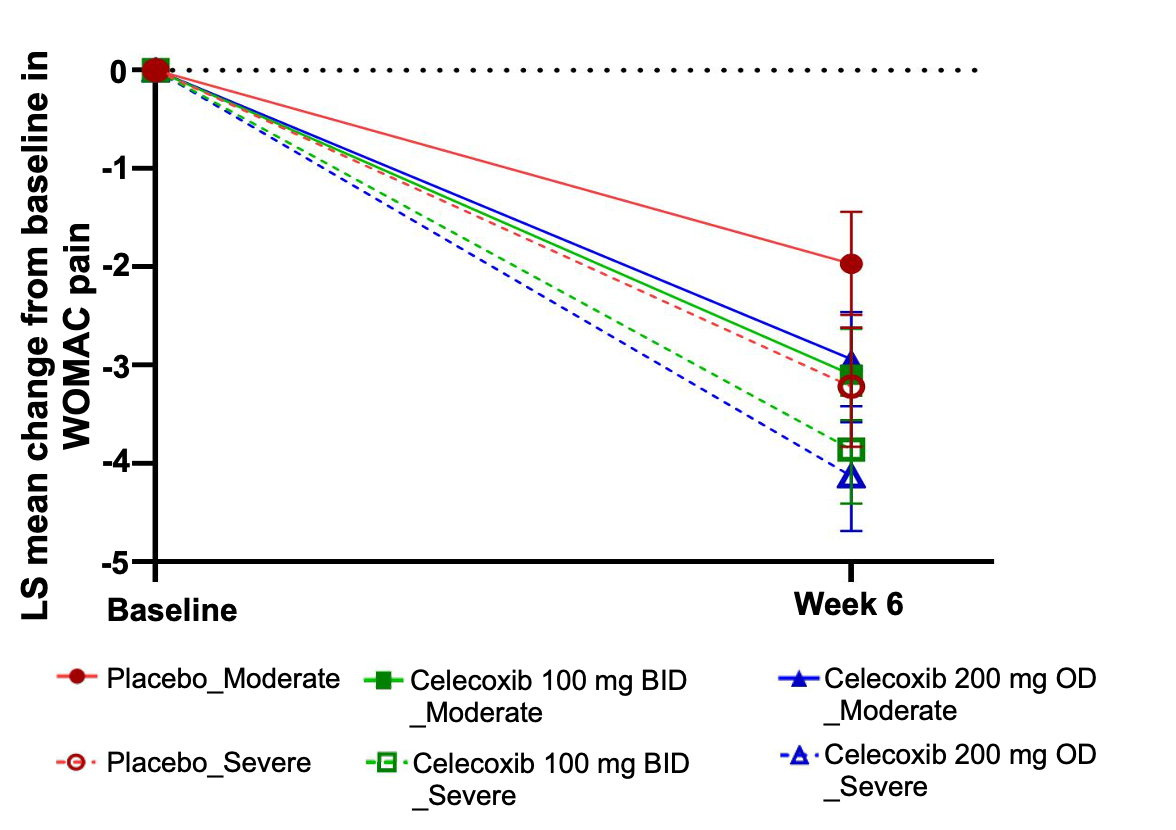
**
